# Letter From The Editor

**DOI:** 10.1017/jme.2022.40

**Published:** 2022

**Authors:** Courtney McClellan

On a recent visit to Los Angeles, California, I walked past a mobile AIDS testing site — a van from the local health department with staff offering help and services. This was a remarkably tangible example of one of the key issues discussed throughout this issue of *JLME*: access. Access to education, access to prevention strategies, access to medication, access to clinical services…. Even though urban areas, like Los Angeles, tend to have more resources than do rural areas, vulnerable populations throughout the U.S. continue to struggle to get tested for HIV, get medication, pay for it and the lab costs, and adhere to the medication. Still in 2022, major barriers to treatment remain, and change is needed.

We at *JLME* are proud to be a small part of this change with “Financing and Delivering Pre-Exposure Prophylaxis (PrEP) to End the HIV Epidemic.” Guest edited by Jeremiah Johnson, Amy Killelea, Derek T. Dangerfield II, Chris Beyrer, and Joshua M. Sharfstein, this issue details a proposal for a national PrEP program and outlines practical strategies to achieve this goal. The focus is on expanding “access, equity, and health” for those vulnerable individuals whose PrEP use is under-utilized, inconsistent, or non-existent. Specific approaches include improved access to laboratory services and pharmacies, telehealth programs, better access to generics, and more community-based programs. The authors of this issue also discuss how a national program could finance and distribute generic PrEP medications — as the government did for the COVID-19 vaccines — and how it could cover the costly lab services for the uninsured and those on Medicaid. Other considerations discussed in the issue include how the high cost of PrEP medications has impacted accessibility and adherence, how adaptability must be a key component of a national program, how addressing the racial and gender disparities should be prioritized, how Medicaid and managed-care organizations could expand access for low-income individuals, and how state and local health departments can take a more active role in increasing PrEP uptake in their communities. Certainly, implementing a new national program will take time, but the next chapter in the fight to end the HIV epidemic will create lasting health equity for all, and it starts in the following pages.

